# Biomechanical analysis of total arch maxillary distalization using infrazygomatic crest miniscrews: a finite element analysis study

**DOI:** 10.1186/s40510-024-00509-3

**Published:** 2024-03-11

**Authors:** Alessandro Schwertner, Renata Rodrigues de Almeida-Pedrin, Thais Maria Freire Fernandes Poleti, Paula Oltramari, Ana Cláudia Ferreira de Castro Conti, Flávio Augusto Cotrim-Ferreira, Guilherme de Almeida, Carlos Flores-Mir, Marcio Rodrigues de Almeida

**Affiliations:** 1 Post-Doctorate Student, Department of Orthodontics, University of North Paraná, UNOPAR, Londrina, PR Brazil; 2 Department of Orthodontics, University of North Parana, UNOPAR, Londrina, PR Brazil; 3 Instituto Vellini, São Paulo, SP Brazil; 4 Department of Pediatric Dentistry and Orthodontics, School of Dentistry, Federal University of Uberlândia, Uberlândia, MG Brazil; 5 Division of Orthodontics, University of Alberta, Edmonton, AB Canada

**Keywords:** Finite element analysis, Total arch distalization, Temporary anchorage devices (TADs)

## Abstract

**Aim:**

To evaluate the maxillary incisors and canine’s immediate movement tendency using three different power arms (PA) height levels during total arch maxillary distalization supported on infrazygomatic crest (IZC) miniscrews according to finite element analysis (FEA).

**Methods:**

Three finite element models of the maxilla were developed based on CBCT imaging of a teenage male patient presenting a Class II Division 1 malocclusion in the early permanent dentition. Maxillary complex, periodontium, orthodontic accessories, IZC miniscrews and an orthodontic wire were digitally created. The PAs were placed between canines and lateral incisors and projected at 4, 7, and 10 mm height distances. After that, distalization forces were simulated between PA and IZC miniscrews.

**Results:**

The anterior teeth deformation produced in the FEA models was assessed according to a Von Mises equivalent. The stress was measured, revealing tendencies of initial maxillary teeth movement. No differences were found between the right and left sides. However, there was a significant difference among models in the under-stress areas, especially the apical and cervical root areas of the maxillary anterior teeth. More significant extrusion and lingual tipping of incisors were observed with the 4 mm power arm compared to the 7 mm and 10 mm ones. The 10 mm power arm did not show any tendency for extrusion of maxillary central incisors but a tendency for buccal tipping and intrusion of lateral incisors.

**Conclusion:**

The maxillary incisors and canines have different immediate movement tendencies according to the height of the anterior point of the en-masse distalization force application. Based on the PA height increase, a change from lingual to buccal tipping and less extrusion tendency was observed for the incisors, while the lingual tipping and extrusion trend for canines increased.

## Background

Skeletal anchorage has transformed the possibilities for Class II malocclusion management in patients with permanent dentition as an alternative to intra- and inter-arch molar distalization approaches [1]. Several locations where skeletal-based screws can be inserted within the oral cavity allow multiple force systems to be applied [2]. Despite being widely used, inter-radicular mini-screws (MS) have shown significant limitations, such as a higher failure risk, a potential for injuring root surfaces, and interference in the path of tooth movement [3, 4]. Inserting MS in areas of extra-alveolar thick cortical bone, such as the infra zygomatic crest (IZC) and the mandibular buccal shelf (BS), minimizes the risk of injuring tooth roots and reduces failure rates when compared to conventional inter-radicular MS. Additionally, it allows for more unrestrained sagittal tooth movement [5–8].

Recent studies have sought a better understanding of the biomechanical effects of total maxillary arch distalization supported by skeletal anchorage devices. They have focused on the potential for molar distalization, the amount and direction of the incisor’s tipping, occlusal plane changes, facial profile effects, and transverse changes resulting from this mechanics’ type [9–11]. The height of the anterior support hook and the distance from the MS head to the occlusal plane are crucial to designing a force system that meets occlusal and esthetic goals [12].

Biomechanical finite element studies are often used because they allow in vitro assessment of immediate tension and stress in digital models. They can be instrumental in assessing skeletal anchorage MS-based anchorage, thus allowing us to find different tendencies for anterior teeth movement [13–15].

The present study uses a finite element analysis (FEA) model to assess immediate teeth movement during total maxillary arch distalization. IZC MS was used with different anterior hook-support height levels. It was hypothesized that longer or shorter hooks produce different intrusion, extrusion, and lingual tipping responses particular to each anterior tooth. Such knowledge would be helpful when developing appropriate treatment planning.

## Materials and methods

Ethical approval was obtained from *the University* and registered at the Clinical Trials Registry. We selected a CBCT scan of a male patient (16y 5 m old) with Class II division1 malocclusion. A significant maxillary dentoalveolar protrusion component was identified. The CBCT scan was taken with teeth in maximum intercuspation position and lips at rest. An OP300 MAXIO CBCT was used (13 × 15 cm FOV, 40 s, 120 kV, 36 mA, 0.3 mm voxel).

FEA was applied to assess the immediate biomechanical behavior of maxillary incisors during an in vitro* en masse* maxillary arch distalization. This was done by virtually applying the force through an IZC MS to a PA positioned at three different height levels (4 mm, 7 mm, and 10 mm) between the maxillary lateral incisors and canines. The CBCT scan in DICOM format was exported to Mimics software 18.0 (Materialise, Leuven, Belgium) to have the outer anatomical traits lined off. Subsequently, the maxilla was segmented from the incisal edge of teeth to the zygomatic bone height. Structures forming the maxilla and the periodontium, particularly teeth and surrounding bone tissues, were simulated using image density thresholding [16, 17]. Boolean operations superimposed a 0.2-mm thick periodontal ligament layer around teeth roots [18, 19]. After segmentation, the three-dimensional triangle-based surface of the maxillary structure was saved into STL format.

Concurrently, 4 mm, 7 mm, and 10 mm height power arms and a 0.19 × 0.25-inch arch were designed with 3-Matic software 18.0 (Materialise, Leuven, Belgium) and saved into STL format. STL files with images of tubes and MBT brackets were provided by ID-Logical (São José do Rio Preto, SP, Brazil). IZC mini-screw STL file was supplied (12 × 2-mm ref. 5593). The latter was placed with the screw head 11 mm above the mesiobuccal cusp of the second molar. All STL files were imported into MSC software (Santa Ana, CA, USA) to form a volumetric tetrahedral element mesh. This mesh was then imported to an FEA software package (MSC Marc/Mentat, MSC Software—MSC Software Co, Los Angeles, CA, USA) for structural analysis.

Linear and three-dimensional elastic analyses were carried out through geometry for the anatomy of maxillary teeth and surrounding cortical and trabecular bones. Nodes were used for the bones and MS system to ensure smooth interface contact. On the top of the bone structure, except for palatal bone, nodes were rigidly fixed in the *x* (horizontal), *y* (vertical), and *z* directions (transverse). The top of the maxillary bone was considered fixed due to its fibrous articulation with the different facial bones through the circummaxillary sutures.

**Fig. 1:**
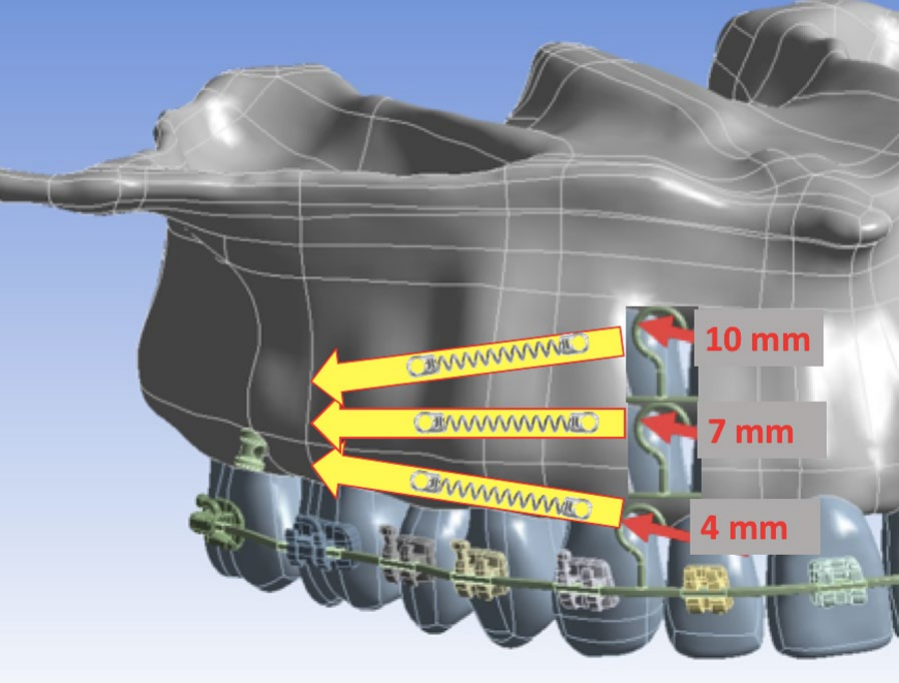
The representative model of each maxillary resultant distalization force used from different power arm heights

For the present study, all materials were considered isotropic, linear-elastic, and homogeneous. Properties of materials applied, such as modulus of elasticity and Poisson’s ratio, were obtained from the literature (Table 1). Interfaces between different structures were considered bonded to avoid relative movement throughout all model interfaces. This means tooth and bone structures cannot be disconnected, thus preventing the rotation of the maxillary section. On the other hand, for the brackets and power arms, contact was considered rigid. Three models were created. On average, models consisted of 537.028 elements and 867.626 nodes (Fig. 1). They varied according to hook heights (A 4 mm, B 7 mm, and C 10 mm). The initial force applied from the IZC MS between the first and second molars to the canine hook was 3.4 N (350 g). A frictional coefficient of 0.15 was adopted between the archwire and the slots.

**Table 1:** Modulus of elasticity (MPa) and Poisson’s ratio (*v*) applied for materials used in the study

Material	Modulus of elasticity (Mpa)	Poisson ratio (*v*)	References
*Properties of materials*			
Mini-screw	114,000	0.34	Mcgraph [20]
Stainless steel appliance	200,000	0.3	Ammoury [21]
Tooth	20,000	0.3	Ammoury [21]
Periodontal ligament	0.68	0.45	Ammoury [21]
Type II medullary bone cortical bone (2 mm) + medullary dense bone	5500	0.3	Tada et al. [22] de Almeida et al. [23]

*Model A* The resultant force was virtually parallel to the occlusal plane.

*Model B* The resultant force was slightly downward towards the posterior region of the occlusal plane.

*Model C* The resultant force was significantly downward towards the posterior region of the occlusal plane.

Data were processed according to the von Misses criteria according to studies by McGrath et al. [20], Ammoury et al. [21], Tada et al. [22], and de Almeida et al. [23] (Table 1) and the trend of initial displacement of incisors, canines and premolars (in mm).

## Results

**Fig. 2:**
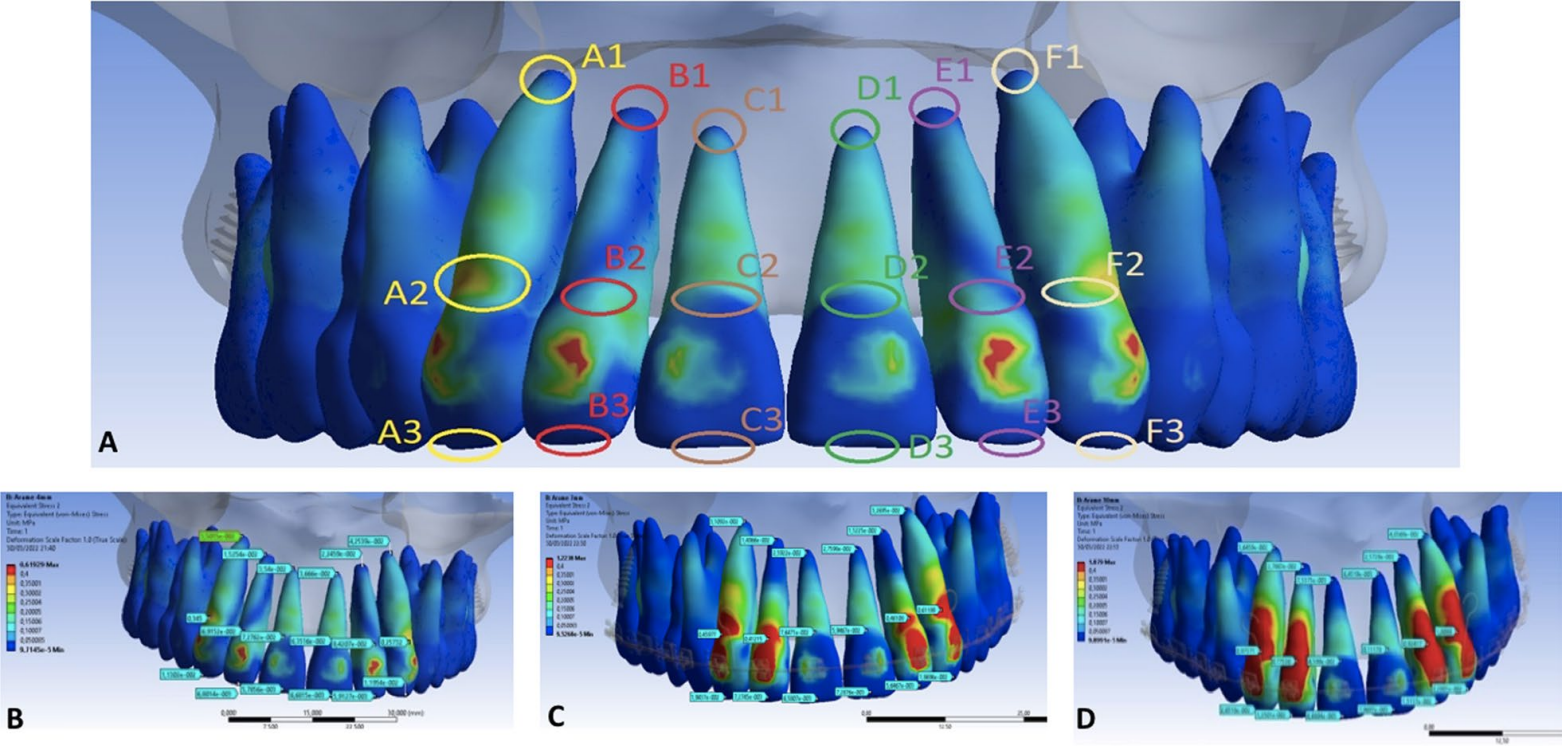
The apical (1), cervical (2), and incisal (3) regions of the anterior teeth and canines **A** showing initial stress according to 4 mm (**B**), 7 mm (**C**), and 10 mm **D** of power arms

Von Misses stress results are expressed in MPa for an initial force of 3.4 N. This applies to analyses carried out for apical (1), cervical (2), and incisal (3) of the anterior teeth and canine regions on both sides, alphabetically ordered from the right to the left sides of the arch (Fig. 2A). The three models of the maxilla with 4 mm (Fig. 2B), 7 mm (Fig. 2C), and 10 mm (Fig. 2D) PA were subjected to *en masse* distalization.

Data obtained from anterior teeth were compared between sides and quantitatively expressed in MPa. Results obtained in each region of each tooth were quite similar for the right and left sides. (Table 2).

**Table 2:** Von Mises stress (MPa) from the right and left sides produced in the apical, cervical, and incisal regions with different height levels of the power arms and 3.4 N of distalization force

Right	4 mm	7 mm	10 mm	Left	4 mm	7 mm	10 mm
A1	0.035	0.031	0.036	D1	0.036	0.027	0.006
A2	0.345	0.459	0.974	D2	0.063	0.059	0.112
A3	0.011	0.019	0.026	D3	0.006	0.007	0.008
B1	0.015	0.015	0.027	E1	0.022	0.015	0.025
B2	0.069	0.412	0.775	E2	0.084	0.461	0.924
B3	0.007	0.007	0.012	E3	0.006	0.005	0.015
C1	0.035	0.025	0.007	F1	0.042	0.032	0.040
C2	0.076	0.076	0.046	F2	0.257	0.612	1.008
C3	0.006	0.006	0.006	F3	0.012	0.018	0.023

**Fig. 3:**
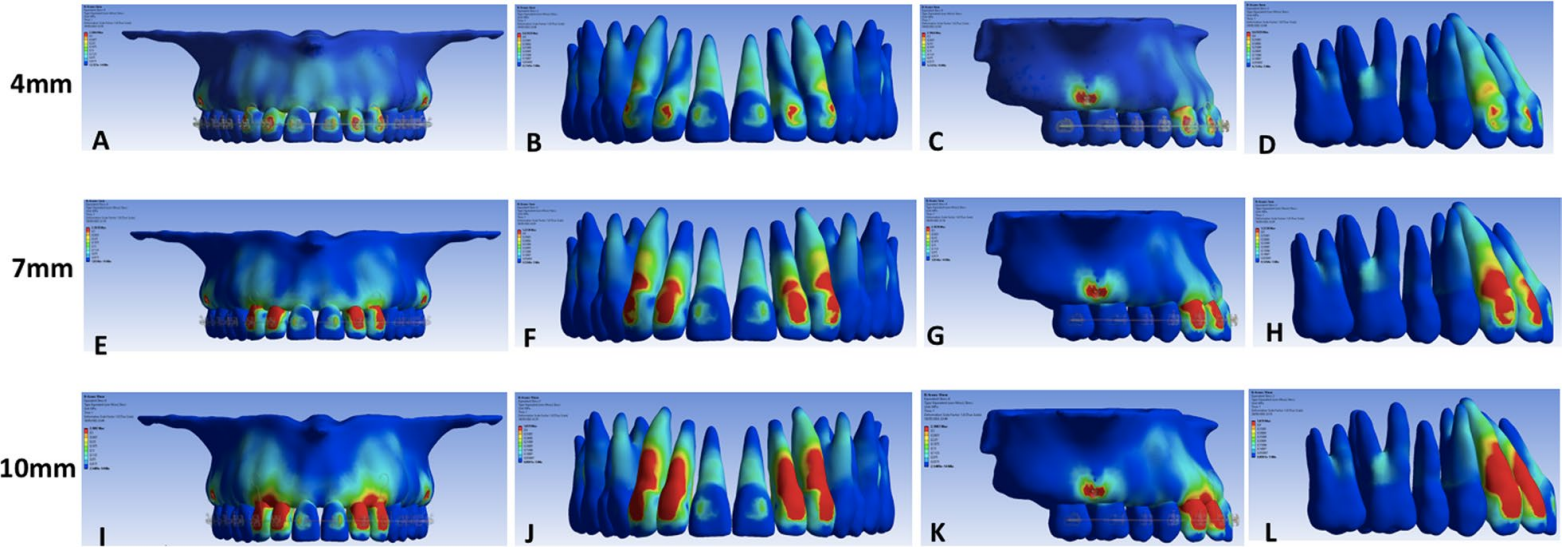
Frontal and lateral views of alveolar bone and teeth according to Von Mises stress produced by force applied for *en masse* total arch distalization using 4-mm (**A**–**D**), 7-mm (**E**–**H**), and 10-mm **I**–**L** power arms

Von Mises stress in the region of IZC MS was similar for the three models (Fig. 3C, G, and K) regardless of the height level of the PA. There were qualitative stress differences (MPa) among models in the apical, cervical, and incisal regions between anterior teeth and canines (Fig. 3).

*Model A* The 4-mm power arm shows more significant stress in the apical region (1) of central incisors both in the bone ridge (Fig. 3A) and the root (Fig. 3B) in comparison to the other areas and teeth. The cervical (2) region of lateral incisors and canines showed slight deformation. The stress coefficient could not be observed in the incisal region (3), particularly in central incisors (Fig. 3B and D).

*Model B* The 7-mm power arm shows lower stress in the apical region (1) of central and lateral incisors. However, there was a significant increase in stress in the cervical area (2) of lateral incisors and canines where MPa (Table 3) and color gradually increased and became more evident (Fig. 3E–H). No deformation was observed in the anterior teeth's incisal region (3).

**Table 3:** Displacement measurements were obtained from the incisal and apical areas of maxillary right incisors and canines according to the X, Y, and Z axes

Area	Group								
	4 mm			7 mm			10 mm		
	Axis								
	*X*	*Y*	*Z*	*X*	*Y*	*Z*	*X*	*Y*	*Z*
A1	0.0000002698	− 0.0000009231	− 0.0000014503	0.000031746	0.00002962	0.000030389	− 0.000060692	0.00002962	0.000010304
B1	− 0.0000067980	− 0.0000066621	− 0.0000049834	0.000025439	0.000026814	0.00003012	7.2229E−06	0.000014261	0.000021989
C1	− 0.0000120860	− 0.0000154250	− 0.0000194340	− 4.7831E−06	− 7.3802E−06	− 9.9725E−06	− 0.00001107	− 0.000012746	− 0.000014583
A2	0.0000812800	0.0000536540	− 0.0000053641	0.0010041	0.00077952	0.00054246	− 0.00042344	0.00077952	− 0.00016278
B2	0.00035556	0.0001411300	0.0000167250	0.00064549	0.000070396	0.00054222	− 0.00031297	0.000070396	− 0.00016204
C2	0.00046657	0.0000703960	− 0.0001478400	0.00053841	0.0007023	− 0.00044699	− 0.0002946	− 0.00037941	0.00054847

*Model C* The 10-mm power arm shows no deformation in the central incisors’ apical region (1). Neither teeth (Fig. 3J) nor the alveolar bone ridge (Fig. 3I) presented deformations. Nevertheless, stress increased in lateral incisors (Fig. 3J)**.** In the cervical region (2), there was a more significant increase in stress qualitatively and progressively observed (Fig. 3J–L) for lateral incisors and canines.

Values expressed in MPa according to von Mises yield criterium were significant when the power arms' different height levels were assessed, showing a magnification of stress in lateral incisors and canines progressively as the height of the power arm increased. This particularly applies to areas around cervical regions and the cervical third of their respective root.

**Fig. 4:**
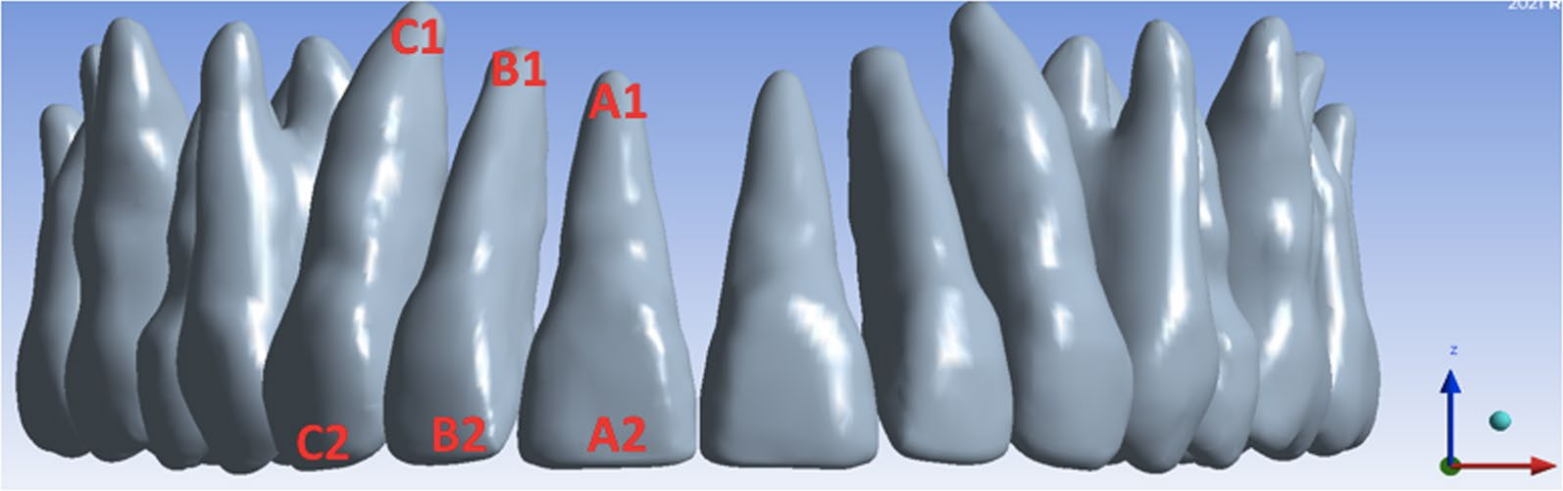
Measurement areas used to obtain the incisal and apical displacement of maxillary right incisors and canines according to the *X*, *Y*, and *Z* axis

Results reveal the tendency for initial movement of incisors and canines differs according to the height of the power arms used for retraction. (Table 3) The trend for distal direction was similar for premolars in the three models assessed (Fig. 4).

**Fig. 5:**
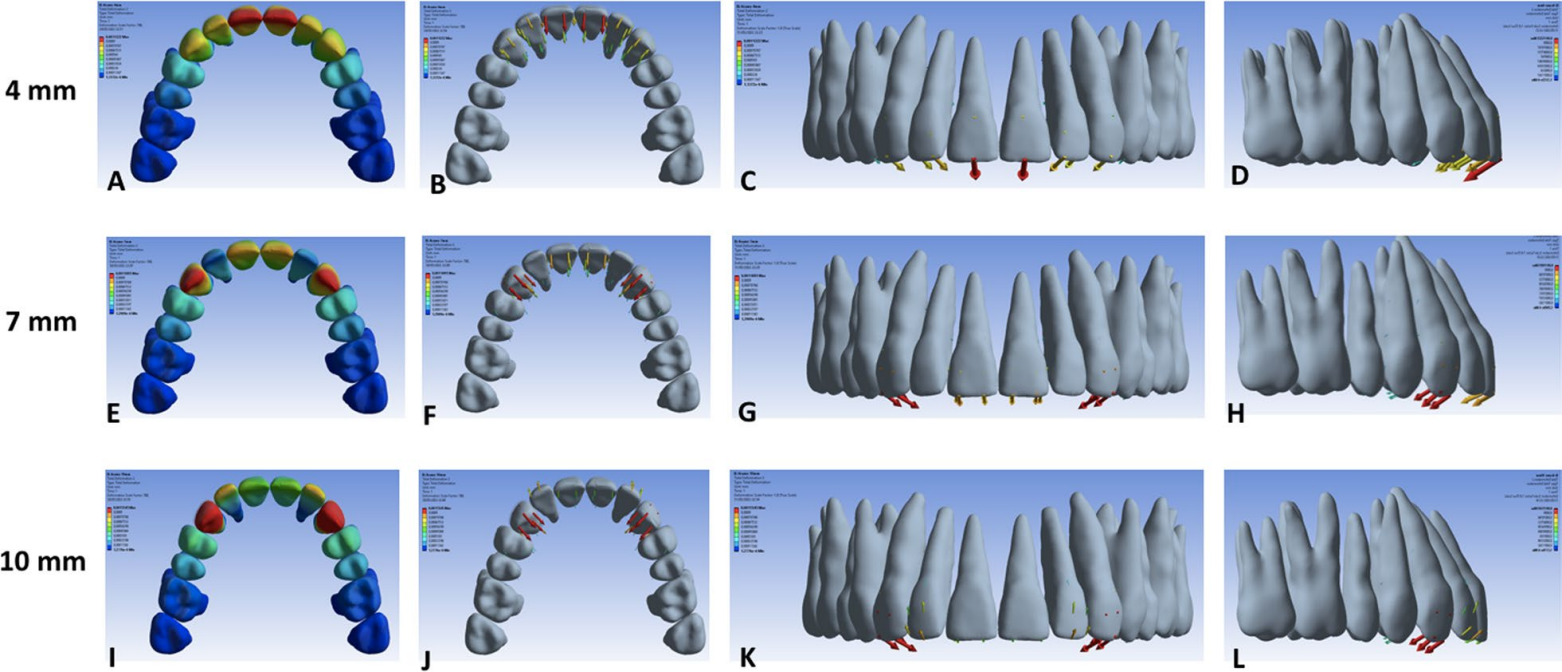
Occlusal (**A**–**B**, **E**–**F**, **I**–**J**), frontal (**C**, **G**, **K**), and lateral **D**, **H**, **L** views of initial displacement tendency from (1) 4 mm, (2) 7 mm, and (3) 10 mm, according to Von Misses equivalent

*Model A* Distalization mechanics with a 4-mm PA showed a predominant tendency for movement of central incisors crown downward (extrusion) and backward (lingual) (Fig. 1B, C), promoting an initial uprighting of 3.42°. The tendency gradually decreased as teeth were assessed in the posterior direction. (Fig. 5A–D).

*Model B* Central incisors reduced the tendency for extrusion and lingual tipping with an initial uprighted movement of 2.67º (Fig. 5F–H). However, there was a tendency for the movement of canines backward and downward (Fig. 5E–H).

*Model C* Examination of the incisors crown revealed a tendency for upward and forward crown movement, with an initial forward displacement of the central incisors' long axis in 1.27ׄ° (Fig. 5J–L). For canines, backward and downward movements remain strong. (Fig. 5I–L).

In the region of central incisors, the shortest PA tends to extrusion and lingual tipping (Fig. 5ª–D). It gradually decreases with 7-mm (Fig. 5E–H) and 10-mm (Fig. 5I–L) PAs. The only model with a tendency for intrusion and buccal tipping in the anterior region occurred when the PA had a height of 10 mm. This was found mainly in the central incisor crown (Fig. 5C, J–L). For canines, an increase in the tendency for lingual and extrusive movements as the height level of the PA gradually increased was clear, starting with the 4 mm model, followed by the 7 mm model, and achieving the highest values with 10 mm (Fig. 5).

## Discussion

There are several options to produce posterior teeth *en-masse* distalization [24, 25]. Assessment of anchorage type*, en-masse* teeth distalization anatomical movement limitations, overjet, and overbite implications are necessary. Moreover, the force vector must be determined to allow resultant favorable forces and prevent undesirable teeth movements [1, 9, 18]. The present in vitro study assesses different PA height levels for *en-masse* maxillary arch distalization to better understand the influence of lines of action of the force on the *en-masse* movement of maxillary teeth [10–13, 24, 26]. This study supports using IZC miniscrews for *en-masse* distalization mechanics as an effective approach for Class II correction with power arms placed mesial of canine. In cases of Class II malocclusion, such as division 1 with an increased overjet and overbite, 10-mm PA associated with the line of the force vector above the center of resistance of anterior teeth allows for the intrusion of anterior teeth and for preventing lingual torque of tooth crown [15, 17], thus favoring malocclusion correction when bite deepening is not sought. Similar results were previously reported with similar mechanics using an 8-mm PA [13].

Absolute anchorage with MS has been widely used [1, 3, 5, 6, 12] as it provides a biomechanically solid anchorage for teeth movement, thus preventing unnecessary teeth displacement and decreasing overall treatment time when compared to intra-arch distalization when the posterior segment is moved first and then the anterior segment [2, 3, 5, 26]. *En-masse* distalization of posterior teeth with IZC MS can be done without changing the screw position [1, 3, 12]. This study used IZC MS heads placed 11 mm above the mesiobuccal cusp of the maxillary second molar, which is a clinically favorable location for screw placement. No significant clinical differences were previously reported between distances 11 mm, 13 mm, and 15 mm as ideal regions for screw placement [11].

FEA studies have been developed to assess theoretically resultant forces obtained by different force vectors, deflection properties of orthodontic alloys, and tooth movement [13–15]. these studies provide a deeper understanding and greater trust in the initial resultant theoretical forces produced when their results are clinically considered. For the method to be used, data about deformations produced and tendencies for movement of teeth are standardized and assumed. It is also important to note that the resultants only refer to the immediate teeth movement stage. Sequential data are needed for each one of the other movement stages to get a more comprehensive view of the phenomenon [9, 13, 24]. This study is based on results obtained only during the immediate movement phase (initial distalization).

A previous FEA study assessed how different force angles applied from the posterior center of resistance produce tooth movements during *en masse* mandibular distalization [9, 24]. Stainless steel archwires varied in thickness (0.016 × 0.022-inch and 0.018 × 0.025-inch), and the force applied was 3N (306 g). Results revealed force vector angles could control the movement of the entire mandibular arch, and slot play between the arch and bracket slot can lead to tooth tipping. The elastic deflection of the wire induced lingual inclination of the anterior teeth, which increased based on the force direction to the occlusal plane.

Similar teeth movement tendencies were found clinically in a prospective clinical trial [10]. IZC screws were inserted 15 mm high relative to the occlusal plane. Total arch maxillary distalization (350 g) was carried out with a 4-mm power arm on 0.017 × 0.025 beta-titanium alloy. This resulted in lingual tipping of 13.42°, anterior teeth extrusion, and posterior teeth intrusion. The force amount used (350 g) and the steel arch (0.019 × 0.025) thickness should be considered when designing this clinical approach [13].

FEA has already been conducted [13] with PA height levels varying from 0 mm, 4 mm, to 8 mm. They were inserted between lateral incisors and canines for *en masse* full maxillary arch distalization with IZC MS as anchorage. Results revealed that incisors tend to extrude when the force system is applied below the center of resistance (0 mm and 4 mm PA)***.*** A prospective clinical trial [10] reported similar findings with an occlusal plane clockwise rotation. In the present study, model A’s PA height (4 mm) showed a tendency for lingual tipping and extrusion in the anterior region. Model C had a higher PA (10 mm) than that used in other studies [10, 13] and showed a tendency for intrusion or even buccal tipping of anterior teeth, particularly lateral incisors. Our results were similar to those of Kawamura et al. [27] for the incisor behavior. However, in our study, the evaluations were restricted to the initial movement tendency of the upper anterior teeth; the height of the hooks was varied, and they were positioned on the mesial surface of the canines and not on their distal surface.

As we understand it, results obtained by in vivo or in vitro studies for *en masse* distalization mechanics with IZC MS might present variations such as the height of screw insertion [10, 11], the amount of force applied [1, 3, 13], the type and thickness of orthodontic archwire alloys [10, 14, 25], hook height [10, 13, 26], and their position mesial of canines or mesial of lateral teeth [10, 13, 14, 26].

In a hypothetical case of Class II, Division 1 malocclusion with open bite and proclined maxillary incisors subjected to compensatory treatment, using a 4-mm PA for anterior retraction might lead to improved tooth relationship due to the tendency for extrusion and lingual tipping of maxillary incisors. This is the desired result for case finishing, as shown by other studies with similar results and traction force in 0 mm and 4 mm hook lengths [11, 13, 14, 26]. To maintain overjet and overbite, using a 7-mm PA with a force vector near the center of resistance of anterior teeth tends to provide better control of anterior teeth tipping and vertical movements. This study also found a tendency for the canines to incline in a lingual direction as the height of the hooks increased from 4 to 10 mm. This can be explained by three reasons: (a) the greater tendency for the hooks to deflect, (b) the corresponding tendency to shift the area of the most significant deformation from the upper central incisors to the canines, and the corresponding displacement of the extrusion tendency and greater lingual inclination also from the same teeth and in the same direction.

As suggested by Lagravere [28], using a complex mathematical tool like FEA is mainly justified to solve mechanics-based problems where an exact mathematical analysis or solution is not possible. Although a user may get an answer from a simulation, it doesn’t mean the solution is accurate. Answering if the justification for using the analysis is solid and relevant and if it has the potential to solve a clinical problem are limiting factors that must always be considered.

FEA studies provide theoretical support for clinical decisions. Nevertheless, the FEA analysis problem lies in the complexity and computing power needed for analyzing teeth movements after immediate teeth displacements. In other words, most FEA studies focus only on the immediate teeth changes but do not consider the complex biomechanical responses. After that, standardized assumptions are needed in FEA studies. Due to the individual variability in every patient’s malocclusion (physiological response, skeletal and dental anatomical characteristics), the results should be cautiously applied in clinical practice. A further clinical study could also be carried out to validate “clinically” the present in vitro results.

## Conclusions

The maxillary incisors and canines have different immediate displacement tendencies according to the height of the anterior point of the en-masse distalization force application. Based on the PA height increase, a change from lingual to buccal tipping and less extrusion tendency was observed for the incisors, while the lingual tipping and extrusion trend for canines increased.

## Data Availability

Please contact the author for data requests.

## Abbreviations

PA: Power arms
IZC: Infrazygomatic crest
MS: Miniscrews
FEM: Finite element analysis
TADs: Temporary anchorage devices
